# Periacetabular osteotomy for symptomatic hip dysplasia in middle aged patients: does age alone matter?

**DOI:** 10.1007/s00402-023-05160-x

**Published:** 2023-12-22

**Authors:** Vincent J. Leopold, Christian Hipfl, Carsten Perka, Sebastian Hardt, Luis Becker

**Affiliations:** Center for Musculoskeletal Surgery, University Hospital Berlin, Charité-Universitätsmedizin Berlin, Charitéplatz 1, 10117 Berlin, Germany

**Keywords:** Developmental dysplasia of the hip, Periacetabular osteotomy, Middle-aged patients, Patient outcomes

## Abstract

**Background:**

Conflicting evidence exists regarding outcomes in middle-aged patients undergoing periacetabular osteotomy (PAO) for symptomatic developmental dysplasia of the hip (DDH).

**Aims:**

To compare patient reported outcomes (PROMs) of middle-aged PAO patients with younger patient groups.

**Methods:**

Retrospective analysis of prospectively collected data of PAO patients between 01/2015 and 06/2017 at a single orthopedic university center with a primary diagnosis of symptomatic DDH. The cohort was divided into four age groups and compared: < 20, 20–30, 30–40 and > 40 years. Joint function was assessed using iHOT-12, mHHS and SHV. Activity level was assessed using UCLA Activity score. Patient satisfaction and pain were assessed on the numerical rating scale 0–10. Conversion rates to THA were assessed.

**Results:**

Out of 202 PAOs, 120 cases with complete data were included. Mean follow-up was 63 months (range 47–81 months). Eighteen patients were < 20 years old, 54 were 20–30 years, 37 were 30–40 years, 11 patients were older than 40. No significant differences were observed for preoperative or postoperative iHOT-12 (*p* = 0.898; *p* = 0.087), mHHS (*p* = 0.878; *p* = 0.103), SHV (*p* = 0.602; *p* = 0.352) or UCLA (*p* = 0.539; *p* = 0.978) between groups. Improvement deltas were also not significantly different for all PROMs. Postoperative patient satisfaction was similar between groups (*p* = 0.783).

**Conclusion:**

Patients with symptomatic DDH may benefit from PAO even at middle age with similar outcomes and pre- to postoperative improvements as younger age groups. Indication should be based on biological age and preoperative joint condition rather than age.

## Introduction

Developmental dysplasia of the hip is the leading cause of secondary osteoarthritis of the hip [1–5]. If diagnosed in time periacetabular osteotomy (PAO) is the treatment of choice in patients with symptomatic acetabular hip dysplasia with good long-term results [6–9]. Through defined osteotomies, the acetabulum is completely detached from the pelvis and then three-dimensionally reoriented, thus improving biomechanics of the joint and avoiding pathologic contact pressure [10, 11]. However, in order to achieve the best possible treatment success, accurate patient selection suitable for this procedure is essential. In addition to the precision of acetabular reorientation, various factors predicting treatment success have been identified. The patient ideally has no or only minor degeneration of the hip joint with a degree of osteoarthritis ≤ 1 according to Tönnis, good joint congruency, and no signs of (sub) luxation [7, 9, 12, 13]. Increasing patient age at the time of surgery has also been defined as a negative outcome predictor in some studies [6, 7]. In these studies, objective criteria such as conversion rate to THA or osteoarthritis progression were used to assess outcome [7, 9, 13, 14]. However, for the individual patient, the impact of surgery on joint-specific function and quality of life may be more important for satisfaction with surgery than the abstract metric of conversion. While some authors consider the ideal PAO patient to be under 35 years of age, others consider 40 years of age to be a relative contraindication for surgery [7, 9, 10]. Little evidence exists on the extent to which such middle-aged patients benefit from PAO in terms of function, quality of life, and satisfaction, and whether differences exist compared to younger patient groups. Therefore, the aim of the presented study was to compare outcomes between different age groups and thus assess the influence of age on patient reported outcomes and patient satisfaction after periacetabular osteotomy. The null hypothesis was that middle-aged patients would benefit from PAO to the same extent as younger patients when well selected.

## Methods

### Study cohort

We performed a retrospective study of prospectively collected data from our institutional PAO database. Approval of the local ethics committee was obtained beforehand (EA1/052/21). Included were patients who underwent PAO between January 2015 and June 2017 at a single orthopedic university center with a primary diagnosis of symptomatic developmental dysplasia of the hip (DDH) based on defined parameters as well as complete information on preoperative and postoperative hip function at follow-up and signed informed consent. Excluded were patients treated with PAO for indications other than symptomatic DDH, osteoarthritis grade > 1 according to Tönnis, prior surgery on the ipsilateral joint or incomplete data.

Four different age groups were defined with patients younger than 20, between 20 and 30, between 30 and 40, and older than 40 at the time of surgery.

DDH was diagnosed from standardized standing anteroposterior radiographs. All hips had ≥ 1 radiographic abnormality, including a lateral center–edge angle (LCEA) according to Wiberg of < 25°, acetabular inclination according to Tönnis (AI) of > 10°, and a femoral head extrusion index (FHEI) according to Heyman and Herndon of > 26%. Femoral head congruency was determined preoperatively via functional radiographs in 30° of abduction and was good in all hips. All hips had osteoarthritis grade ≤ 1 according to Tönnis.

### Surgical procedure

All PAOs were performed according to the Ganz technique as previously described [15]. An anterior approach was used, and acetabular reorientation was achieved under fluoroscopic guidance. A normalization of the LCEA to 30–35°, AI below 10° and an FHEI of between 10 and 25% were targeted intraoperatively.

### Data collection

Hip function was assessed using the modified Harris Hip Score (mHHS), International Hip Outcome Tool 12 (iHOT-12) and Subjective Hip Value (SHV). While the latter have been previously found suitable in the context of hip joint preservation surgery [16, 17], the mHHS is predominantly used in the context of hip arthroplasty. Nevertheless, it was additionally used in the presented study to allow comparability with the few previous studies that have assessed outcome after PAO in middle-aged patients [7, 18]. Patient-reported outcome measures (PROMs) were collected at follow-up via a hip-specific questionnaire. Patient satisfaction and pain were assessed via Numerical Rating Scale (NRS). Activity level was assessed using the UCLA Activity Score (UCLA). PAO failure was defined as conversion to total hip arthroplasty. Patients were contacted for follow-up by mail and phone.

### Statistics

Statistical analyses were performed using SPSS Version 27 (IBM Corporation, New York, USA). For statistical analysis of pairwise comparison of paired data Wilcoxon-rank sum test was used. For comparison between more than two groups, Kruskal–wallis test with post hoc testing with Bonferroni correction was used. Pearson's chi-square test was executed for testing distribution differences of categorical data. The significance level was set at *p* < 0.05 for all tests.

### Source of funding

No funding was received for this study.

## Results

### Demographics

A total of 202 consecutive hips in 183 patients who underwent PAO between 01/2015 and 06/2017 were included. Six hips were excluded due to primary diagnosis of acetabular retroversion and eleven due to insufficient pre- or postoperative imaging. Sixty-five hips were excluded due to missing follow-up data. Thus, we included a total of 120 PAOs in 103 patients.

Eighteen patients were under 20 years old at the time of surgery, 54 between 20 and 30 years, 37 between 30 and 40 years, 11 patients were older than 40. Mean follow-up was 63 months (range 47–81 months). Demographics of the different groups are given in Table 1.

**Table 1:** Patients’ demographics

	< 20	20–30	30–40	> 40	*p*
*N* =	18	54	37	11	
Age	17.3 (± 1.4)	24.7 (± 2.9)	33.7 (± 2.8)	42.3 (± 1.8)	** < 0.001**
BMI	21.2 (± 1.6)	24.1 (± 5.3)	25.6 (± 4.0)	24.6 (± 4.8)	**0.008**
Female: Male	17:1	48:6	27:10	10:1	0.094
Tönnis osteoarthritis grade (0/1)	18/0	28/26	5/32	0/11	** < 0.001**

Values are presented as mean and standard deviation. Significance level was set at *p* < 0.05. Intergroup comparison was performed by Kruskal–wallis test for age and BMI. Chi-square test was performed for sex and Tönnis arthrosis grade. Significant values are marked in bold.

### Effect of PAO on radiological parameters

In all groups, significant improvement in femoral head coverage was achieved by PAO as presented in Table 2.

**Table 2:** Effect of PAO on radiological parameters

	< 20 years			20–30 years			30–40 years			> 40 years		
	Pre-OP	Post-OP	*p*	Pre-OP	Post-OP	*p*	Pre-OP	Post-OP	*p*	Pre-OP	Post-OP	*p*
LCEA	16.4 (± 4.3)	30.3 (± 6.0)	** < 0.001**	15.0 (± 7.0)	29.8 (± 6.5)	** < 0.001**	16.2 (± 5.3)	29.2 (± 6.0)	** < 0.001**	20.6 (± 3.9)	29.2 (± 4.2)	**0.003**
TA	15.3 (± 6.6)	0.6 (± 8.3)	** < 0.001**	12.8 (± 7.4)	0.2 (± 7.5)	** < 0.001**	13.3 (± 5.6)	2.4 (± 7.3)	** < 0.001**	11.9 (± 6.6)	3.1 (± 4.9)	**0.007**
FHEI	0.24 (± 0.07)	0.09 (± 0.08)	** < 0.001**	0.25 (± 0.09)	0.10 (± 0.10)	** < 0.001**	0.24 (± 0.08)	0.09 (± 0.08)	** < 0.001**	0.19 (± 0.05)	0.09 (± 0.03)	**0.003**

Values are presented as mean and standard deviation. Significance level was set at *p* < 0.05. Intergroup comparison was performed by Wilcoxon-rank sum test. Significant values are marked in bold

### Differences in PROMs pre- and postoperatively

Overall, significant improvements were observed for mHHS, iHOT-12, SHV and UCLA (*p* < 0.001) across the study cohort.

No significant differences between the age groups were detected for PROMs pre- nor postoperatively by intergroup comparison. Consequently, no differences for postoperative function as measured by mHHS, iHOT-12 and SHV were observed. Pre- to postoperative differences in PROMs were also not significantly different for all measures between groups. There was also no significant difference in postoperative patient satisfaction between age groups. For a detailed summary of intergroup comparison, see Table 3.

**Table 3:** Differences in PROMs between the age cohorts pre- and postoperatively

	Pre-PAO					Post-PAO					Delta Pre- Post PAO				
	< 20	20–30	30–40	> 40	*p*	< 20	20–30	30–40	> 40	*p*	< 20	20–30	30–40	> 40	*p*
iHOT-12	40.8 (± 24.4)	41.3 (± 22.2)	42.7 (± 21.3)	38.3 (± 24.1)	0.898	75.7 (± 16.5)	69.8 (± 23.6)	78.2 (± 22.3)	62.0 (± 27.3)	0.087	32.8 (± 25.4)	32.6 (± 21)	39.9 (± 24.1)	37.1 (± 19.5)	0.070
mHHS	49.3 (± 21.9)	41.3 (± 22.2)	42.7 (± 21.3)	38.3 (± 24.1)	0.878	91.6 (± 10.6)	69.8 (± 23.6)	78.2 (± 22.3)	62 (± 27.3)	0.103	42.3 (± 22.6)	30.6 (± 16.6)	38.2 (± 15.8)	34.3 (± 22.4)	0.383
SHV	48.2 (± 24)	42.9 (± 22.7)	41.0 (± 26.5)	40.0 (± 27.1)	0.602	85.8 (± 8.1)	79.0 (± 17.6)	82.2 (± 18)	70.9 (± 24.9)	0.352	40.3 (± 28.5)	35.7 (± 22)	44.1 (± 26.1)	43.6 (± 22.8)	0.419
UCLA	4.4 (± 2.5)	5.1 (± 2.4)	5.3 (± 2.4)	5.4 (± 2.7)	0.539	7.1 (± 2.0)	7.0 (± 1.7)	7.0 (± 1.7)	6.6 (± 2.2)	0.978	2.7 (± 3)	2.3 (± 2)	2.5 (± 1.7)	2.7 (± 1.3)	0.677
Pain	6.8 (± 2.0)	7.2 (± 1.8)	7.5 (± 1.8)	6.9 (± 2.3)	0.575	5.6 (± 2.4)	2.4 (± 2.3)	2.0 (± 1.6)	3.2 (± 2.8)	0.142	5.6 (± 2.4)	4.9 (± 2.4)	5.5 (± 2.0)	3.7 (± 3.4)	0.334
Patient satisfaction						7.8 (± 2.9)	7.8 (± 2.3)	8.2 (± 2.2)	7.2 (± 3.4)	0.783					

Values are presented as mean and standard deviation. Intergroup comparison was performed by Kruskal–Wallis test. Significance level was set at *p* < 0.05.

### Conversion to arthroplasty

Two patients underwent conversion to THA in the follow-up period. No differences were detected between the age groups as presented in Table 4

**Table 4:** Conversion rate of PAO to hip arthroplasty in the age cohorts

	< 20	20–30	30–40	> 40	*p* value
Conversion to hip arthroplasty (yes/no)	0/18	1/53	0/37	1/10	0.200

Conversion rate to hip arthroplasty is presented in count of hips, which were converted to THA in the follow-up time. Conversion rate was evaluated by Chi-Square Test. Significance level was set at *p* < 0.05

## Discussion

While PAO is well established as the treatment of choice for symptomatic hip dysplasia in the prearthrotic state, there are no uniform recommendations for the upper age limit above which PAO should no longer be recommended.

The results of our study showed no significant difference in PROMs and activity level as well as patient satisfaction between the age groups studied in a cohort of 120 PAOs at mid-term follow-up. Preoperative and postoperative values as well as the delta of improvement were comparable in the different groups. Conversion to THA was low with just two cases in the entire cohort one of which occurred in the age group 20–30 and one in the group above 40 years.

In a long-term follow-up study by Lerch et al., in addition to a lower preoperative mHHS and degree of osteoarthritis ≥ 2 according to Tönnis, patient age was identified as a negative outcome predictor associated with higher conversion rates to THA [7]. In our cohort, there was no difference between groups in terms of preoperative mHHS, whereas the older groups were significantly more likely to have an osteoarthritis grade of 1 but not higher. This suggests that if certain indication criteria, such as preoperative function and degree of osteoarthritis, are met, PAO can be indicated even in advanced age and good postoperative function and patient satisfaction can be achieved.

In a study by Zhu et al., 36 middle-aged patients between 35 and 47 years were followed-up at a mean of 5 years and showed good hip survivorship and function as measured using the mHHS concluding that PAO should be indicated in those patients. While the follow-up time was comparable to our study cohort, no comparison was made with younger patients [19].

In a retrospective multicenter study investigating PAO outcomes in patients over the age of 40, Millis et al. assessed hip function and survivorship in 87 PAO cases at a mean follow-up of nearly 5 years. The authors found that at follow-up, 24% of patients converted to THA, but those remaining showed good pain reduction and function as measured using the mHHS. While there was no difference in preoperative function in this cohort studied, the degree of osteoarthritis appeared to be a determinant of outcome with a 27% risk for THA conversion in patients with preoperative Tönnis 2 degree of osteoarthritis [18]. This underlines that preoperative joint condition or biological age rather than the mere age of the patient seems to be of greater importance for the indication. This is consistent with the results of our study in which no patient had a preoperative degree of osteoarthritis > 1 and good function and satisfaction could be achieved even in middle-aged patients with low conversion rates. However, the authors did not compare outcomes with younger patient groups [18].

While such studies have reported good results in middle-aged PAO patients but have not compared these with younger groups [18, 19], other authors recently compared outcome between age groups with a similar grouping as in our study with conflicting results [20, 21]. In a multicenter study reported by Muffly et al., 391 PAO patients were assessed with the same age grouping as in our study design and improvement was observed in all age groups at 2-year follow-up. Interestingly, significantly greater functional improvement was observed in the age group above 40 years than in patients < 20 and 30–39 years. On the other hand, no influence of age and degree of osteoarthritis on outcome was observed, and the authors concluded that age alone should not be an appropriate selection criterion. In contrast to our study with a mid-term follow-up, the follow-up in the aforementioned work was relatively short, limiting longer-term conclusions. However, similar to our results, it was shown that with good patient selection, PAO can achieve excellent functional results in middle-aged patients.

The other study with the same age grouping by Franken et al. assessed 86 PAO patients (106 hips) at mid-term follow-up. The authors reported significant improvements in all 4 age groups, but both lower preoperative and postoperative joint-specific function were observed in patients aged 40 years and older. However, the differences between preoperative and postoperative conditions were similar between age groups without significant differences between improvement deltas [20]. This is consistent with the observations of our study, where there was also no difference in functional improvement deltas pre- to postoperatively between the investigated groups.

The contrasting results of the different studies may suggest either concomitant co-pathologies of the hip that impact surgical outcomes or different patient selection, which makes comparability difficult. Degenerative joint changes, which are often associated with increasing age, intra-articular chrondrolabral pathologies or concomitant femoroacetabular impingement probably play a particularly important role. An isolated view of specific influencing factors such as age alone therefore does not appear to be sufficient. Rather, the indication for PAO must be made in a multifactorial concept that considers various other factors in addition to patient age alone [22–24]. In summary, although mixed results of various studies with partly different study designs are available so far, the sole patient age seems to be unsuitable as an absolute indication criterion. A subjective age threshold above 35 or 40 years as a contraindication for PAO, as also found in the literature, thus seems at least questionable [9, 25].

The present study has several limitations. The first limitation of our study is the retrospective study design. A second limitation is the relatively small number of patients in the group over 40 years of age, which may limit power. However, with the comparison between different age groups, this study adds to the scarce evidence on the studied topic. Third, the mHHS is historically more established for the assessment of THA patients, and ceiling effects have been reported in the context of hip preservation surgery. However, its use in this study allows comparison with the few previous studies on the topic and additionally the iHOT-12 was used. A fourth limitation is the relatively large loss to follow-up due to change of contact information. However, we were still able to follow-up a total of 120 patients. Lastly, it must be stated that in the group over 40 years of age, the preoperative radiological parameters indicate milder dysplasia at mean. However, similar correction values were obtained postoperatively in all groups.

## Conclusion

Patients with symptomatic hip dysplasia may benefit from PAO even at middle age with similar outcomes and pre- to postoperative improvements compared to younger age groups, if well selected. The indication should be based on the biological age of the patient and the preoperative joint condition rather than mere age as part of a multifactorial concept. The results of this study can be useful to orthopedic surgeons in decision making and patient education when considering PAO in middle-aged patients.

## Data Availability

The data analyzed during the current study will be shared by the corresponding author upon reasonable request.

## Declarations

### Level of evidence

III, observational cohort study.
